# PD80 Exploring Key Factors Influencing Patients’ Antidepressant Preferences: Insights From A Multicenter Best-Worst Scaling Survey

**DOI:** 10.1017/S0266462324003337

**Published:** 2025-01-07

**Authors:** Ying Tao, Yanfeng Ren, Shimeng Liu, Yingyao Chen

## Abstract

**Introduction:**

Depression is associated with serious disease burden. Despite the multitude of antidepressant options available, the adherence rate is often low. Accounting for patient preferences can potentially boost adherence to antidepressant medication and elevate patient satisfaction. However, limited evidence exists regarding patient preferences for antidepressant selection. This study aims to elicit patient preferences regarding the benefits, risks, and cost attributes of antidepressants in China.

**Methods:**

A best-worst scaling profile case experiment was conducted using a face-to-face survey administered to patients diagnosed with depression. Patients were recruited from general and psychiatric hospitals. We utilized a multiphase approach that integrated literature review, expert consultation, and best-worst scaling to develop attributes within choice sets. The attributes with each varying across two or three levels encompassed remission rate, sleep disorders, risk of headache or dizziness, risk of gastrointestinal adverse events, risk of liver or kidney injury, and monthly out-of-pocket costs. Each respondent answered seven choice tasks, including a dominant task. Data were analyzed using conditional logit, mixed logit, and generalized multinomial logit models. Subgroup analyses were conducted to explore preference heterogeneity.

**Results:**

A total of 331 participants completed the survey and met the inclusion criteria. Almost all attribute levels were statistically significant. Overall, the most desirable characteristics of antidepressant medications were higher remission rates (80% and 55% rates; p<0.05), lower risk of liver or kidney injury (1% rate; p<0.05), and fewer monthly out-of-pocket costs (CNY100 [USD13.93, EUR12.75]; p<0.05). Risks of gastrointestinal adverse events (60% and 35% rates) and insomnia were the least preferred features. Regarding attributes, efficacy, the risk of gastrointestinal adverse events, and sleep disorders were relatively important factors influencing patient choice. Preferences differed slightly by age, degree of education, personal annual income, and treatments currently received.

**Conclusions:**

Our study suggests that efficacy, gastrointestinal adverse effects, sleep disorders, and treatment costs are critical drivers behind medication choices among patients with depression. Preference heterogeneity also exists regarding individual and therapeutic characteristics, which need more samples and further analyses to identify. These discoveries hold the potential to enrich the shared decision-making process between physicians and patients within healthcare settings.

